# Genetically engineered *Escherichia coli* Nissle 1917 enabling on-site melanin synthesis attenuates radiation enteritis through ferroptosis inhibition and gut microbiota modulation

**DOI:** 10.1016/j.redox.2026.104141

**Published:** 2026-03-25

**Authors:** Chaoqun Lv, Hongqing Li, Xiang Li, Wen Shi, Wenbo Li, Zhenxing Li, Xinyue Hu, Xinxin Liu, Yuanyuan Ai, Zhipeng Wen, Feng Liu, Yi Ru, Haijun Xiao, Jingchao Li, Xiao Chen, Kaijun Liu

**Affiliations:** a Department of Intensive Care Medicine, Army Medical Center of PLA, Daping Hospital, Army Medical University, No.10 Changjiang Branch Road, Yuzhong District, Chongqing, 400042, China; b Department of Nuclear Medicine, Army Medical Center of PLA, Daping Hospital, Army Medical University, No.10 Changjiang Branch Road, Yuzhong District, Chongqing, 400042, China; c Department of Anesthesiology, Stomatological Hospital of Chongqing Medical University, Chongqing, China

**Keywords:** Radiation enteritis, EcN-Tyr(A/C)_1_ microspheres, Melanin, Ferroptosis, gut microbiota

## Abstract

Radiation enteritis (RE) poses a clinically-relevant therapeutic challenge with limited effective interventions. Engineered probiotic drug delivery systems offer innovative strategies for precise treatment of inflammatory disease. However, both the practical efficacy and therapeutic mechanism of engineered probiotic agents for RE alleviation remains largely unclear. Herein, the melanin with natural radioprotective function was applied to modify engineered *Escherichia coli* Nissle 1917 that contains the tyrosinase gene (EcN-Tyr), which were further formulated into orally administrable microspheres (EcN-Tyr (A/C)_1_) with natural sodium alginate and chitosan coatings via microfluidic approach. Notably, EcN-Tyr (A/C)_1_ microspheres could successfully withstand gastric acid and actively target inflammatory lesions in the intestine. Mechanistically, EcN-Tyr (A/C)_1_ microspheres enabled ferroptosis inhibition through reducing lipid peroxidation to protect the host from radiation damage. As a result, EcN-Tyr (A/C)_1_ effectively alleviated radiation-induced intestinal inflammation, and reduced DNA damage. Furthermore, the administration of EcN-Tyr (A/C)_1_ increased the abundance of beneficial bacteria, such as Akkermansia and Ligilactobacillus, while reducing the abundance of harmful bacteria, such as Escherichia-Shigella, clearly indicating the positive effects on the balance of gut microbiota. In summary, EcN-Tyr (A/C)_1_, as a novel probiotic carrier, shows great potential in the treatment of RE, and pioneers new avenues for leveraging natural biomaterials to treat RE.

## Highlights

• Engineered melanin-modified probiotics target and alleviate radiation enteritis.
• EcN-Tyr (A/C)_1_ microspheres inhibit ferroptosis and reduce intestinal DNA damage.
• Treatment restores gut balance by boosting good and reducing harmful bacteria.

## Graphical abstract

**Figure:**
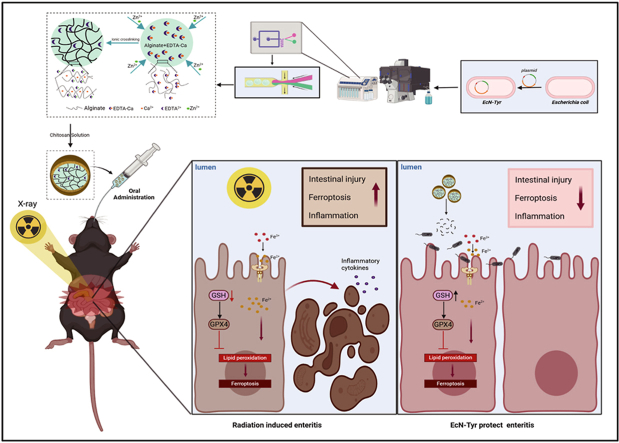

## 1 Introduction

Radiation enteritis (RE) often occurs in various forms of radiotherapy [[1], [2], [3], [4], [5]], due to the large volume and radio-sensitive nature of the intestine [6,7]. The underlying pathological mechanisms primarily involve radiation-induced DNA damage in intestinal mucosal cells, triggering the excessive accumulation of reactive oxygen species (ROS). This in turn activates the ferroptosis pathway via lipid peroxidation, leading to necroptosis of intestinal epithelial cells [8,9]. Concurrently, gut microbiota dysbiosis exacerbates the inflammatory cascade, forming a vicious cycle that amplifies tissue damage [10,11]. The severity of RE depends on both the exposure dose and the duration of radiation. It may lead to significant morbidity, including acute and chronic symptoms. It is estimated that at least 75% of patients experience RE after abdominal or pelvic radiation therapy [12]. Despite the longstanding recognition of radiation-induced gastrointestinal (GI) tract injury, there remains a lack of specific protective agents against RE. Therefore, an effective therapeutic strategy to prevent radiation-induced gastrointestinal injury is essential.

Bacterial-based drug delivery systems have emerged as promising biomimetic platforms to enhance therapeutic efficacy [[13], [14], [15], [16], [17]]. These systems facilitate sustained drug delivery, improve drug stability, reduce dosage and toxicity, enhance targeting capabilities, and potentially integrate drug production and on-site delivery [18]. *Escherichia coli* Nissle 1917 (EcN) is one of the most widely utilized engineered bacteria for the treatment of tumors and gastrointestinal diseases [19,20]. Compared to other bacterial strains, EcN exhibits notable antibacterial and anti-inflammatory properties, regulates intestinal flora, and functions as a facultative anaerobe [6,7]. EcN demonstrates efficacy in mitigating inflammation and preserving barrier integrity in intestinal disorders [21]. Consequently, it has been extensively investigated as a therapeutic strategy for inflammation-related conditions, including inflammatory bowel disease (IBD). Multiple studies indicate that EcN's therapeutic effects are comparable to mesalazine in IBD treatment (with enhanced efficacy observed when used in combination) [[22], [23], [24]]. Nonetheless, the potential therapeutic application of EcN in RE still remains underexplored.

Apart from functioning as a natural carrier and releasing drug cargo slowly through its own colonization ability, another advantage of EcN carriers is that it can express exogenous protein [25]. Melanin, as a biological pigment in human body, is formed by a series of chemical reactions of tyrosine or 3,4-dihydroxy phenylalanine (l-DOPA) and has exceptional biocompatibility and biodegradability [26]. Melanin demonstrates a variety of biomedical applications including ultraviolet (UV) irradiation protection, cancer theranostics and anti-infection therapy [[27], [28], [29]]. Notably, the in vivo radioprotective effect of melanin has been widely validated. It scavenges radiation-induced ROS, inhibits lipid peroxidation and ferroptosis, and even protects intestinal tissues from radiation damage via oral administration [[30], [31], [32], [33]]. However, exogenous melanin suffers from poor targeting, short half-life, and low bioavailability, while in-situ synthesis of melanin by probiotics for sustained RE protection remains underexplored.

Tyrosinase is a copper-containing enzyme, which plays a key role in melanin metabolism. Therefore, we were inspired to fabricate engineered bacteria EcN-Tyr that can be served as endogenous melanin-supplying biofactory and targeted drug delivery system for effective RE treatment.

**Scheme 1:**
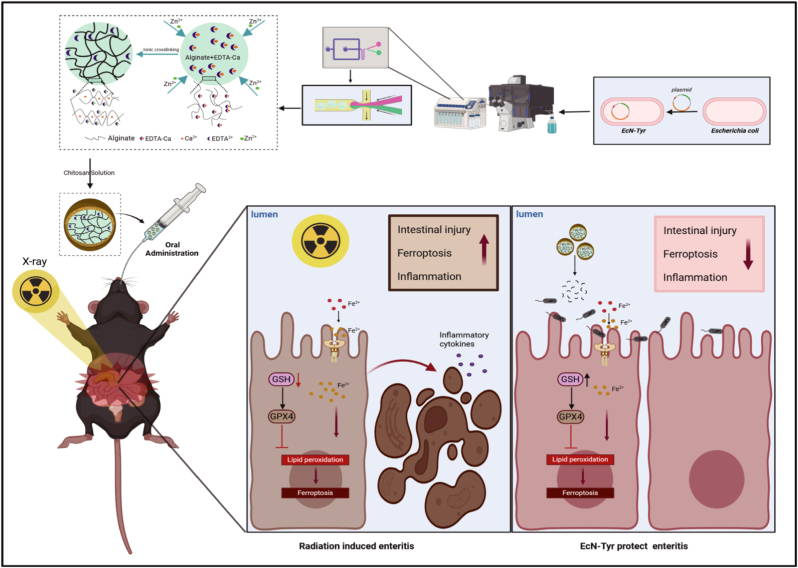
**Engineered probiotics for RE treatment.***Escherichia coli* Nissle 1917 (EcN) was engineered to express tyrosinase, an enzyme that catalyzes melanin synthesis. The resulting recombinant bacteria were subsequently encapsulated using a layer-by-layer assembly of chitosan and sodium alginate, forming (EcN-Tyr (A/C)_1_. Following oral administration in mice, the sodium alginate/chitosan coating substantially enhanced the bioavailability of EcN-Tyr (A/C)_1_ in the gastrointestinal tract. This formulation scavenges ROS to inhibit lipid peroxidation-driven ferroptosis, which alleviates inflammation and restores gut microbiota equilibrium, thereby providing comprehensive protection against RE.

The modification of EcN is crucial for maintaining its viability in harsh gastrointestinal environments. As a natural component extracted from ocean algae and chitin, the chitosan/sodium alginate coating demonstrated a shielding effect in the gastrointestinal tract and enabled targeted delivery to the inflammatory site [34,35]. In this study, to develop rationally engineered bacteria and reveal its therapeutic effect on RE, we constructed EcN-Tyr strains endogenously producing melanin with structural equivalence to the chemically synthesized one. Then, we found that EcN-Tyr shows a protective effect against radiation-induced intestinal damage by inhibiting ferroptosis, alleviating inflammation and restoring microbial homeostasis of gut microbiota. In addition, we used microfluidic technology to prepare oral bacterial microspheres and found that they have better intestinal targeting efficiency and enteritis protective effects after oral administration. Collectively, these results provide a promising therapeutic strategy to protect against RE (Scheme 1).

## 2 Results

### 2.1 Ferroptosis as a key pathogenic driver in RE

**Fig. 1:**
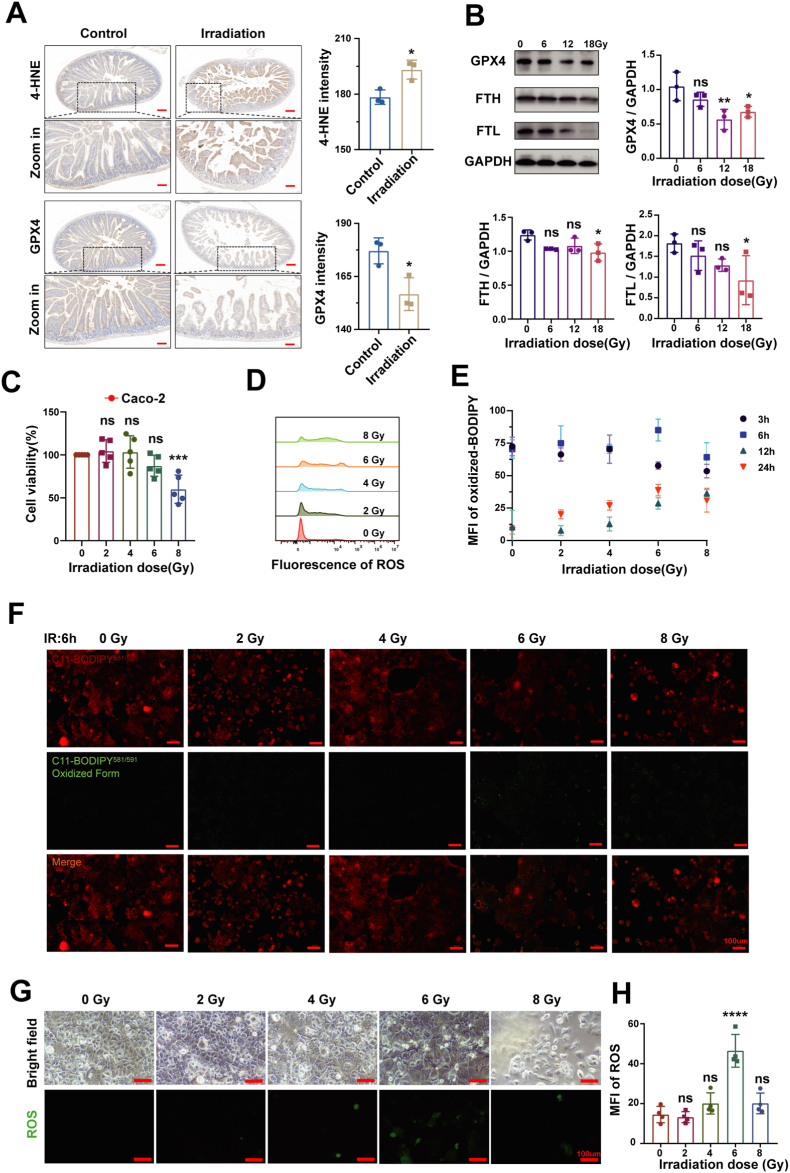
Ferroptosis plays critical role in the pathogenesis of RE. A) Representative immunohistochemical staining of 4-HNE and GPX4 of small intestinal tissue samples from mice with RE, and their normalized expression intensity (relative to non-irradiation), n = 3. Scale bar: 200 μm (top panels) and 50 μm (bottom panels). P values were calculated by using a two-tailed unpaired Students *t*-test. B) Representative Western blotting image and analysis of ferroptosis-related proteins expression in small intestinal tissue of mice (relative to GAPDH) levels, n = 3. C) Cell viability after incremental dose IR exposure (n = 5). D) Representative flow cytometry histogram of ROS (10,000 cells per tube were collected). E, F) Fluorescence images of reduced (red) and oxidized (green) C11-BODIPY in Caco-2 cells after 6 h exposure of IR, along with the quantified results of average fluorescence intensity (n = 5, Scale bar: 100 μm). G, H) Immunofluorescence images of ROS and their mean fluorescence intensity quantification (n = 3. Scale bar: 100 μm). Error bars are presented as mean ± standard deviation (SD). The data were analyzed by one-way ANOVA with Tukey's post hoc test (*P < 0.05, **P < 0.01, ***P < 0.001, ****P < 0.0001).

Accumulating evidence implicates elevated iron levels and lipid peroxidation in the pathogenesis of RE [36]. Immunohistochemical analysis showed a marked increase in 4-Hydroxynonenal (4-HNE), a terminal product of ferroptosis, alongside a significant decrease in Glutathione Peroxidase 4 (GPX4). As a lipid peroxidation product, 4-HNE can induce cytotoxicity and cell death across various cell types [37]. GPX4, one of eight known mammalian glutathione peroxidases, is distinguished from other isoforms by its unique capacity to reduce esterified oxidized fatty acids and cholesterol hydroperoxides [38]. Representative results are shown in Fig. 1A. Together, these findings strongly suggest that ferroptosis contributes critically to the pathogenesis of RE.

To investigate the correlation between ferroptosis and RE, we analyzed the ferroptosis-related proteins in small intestine of mice exposed to ionizing radiation (IR). The expression of GPX4, ferritin heavy chain (FTH), and ferritin light chain (FTL) decreased significantly with increasing IR dose (Fig. 1B), indicating that ferroptosis contributes critically to IR-induced intestinal injury in a dose-dependent manner. The mechanism by which IR downregulates GPX4 remains unclear, though potential explanations involve transcriptional repression or epigenetic silencing mediated by radiation-responsive transcription factors and chromatin-modifying enzymes [39]. Ferritins (FTH and FTL) store and detoxify cytosolic iron, and their downregulation implies an IR-induced disruption of iron metabolism [40]. These sequential alterations revealed a significant inverse correlation between radiation dose and the expression of GPX4, FTH, and FTL (Fig. 1B), supporting a potential mechanistic connection between RE and ferroptosis.

Small intestinal epithelial cells are highly radiosensitive, and their death disrupts the small intestinal barrier, representing a primary pathogenic mechanism of RE [41]. We therefore investigated IR-induced cell death in vitro using Caco-2 cells (human colonic epithelial cells). Radiation exposure significantly reduced cell viability (Fig. 1C) and directly stimulated ROS production (Fig. 1D and G), while also elevating lipid peroxidation levels (Fig. 1F). Upon IR exposure, cells exhibited an immediate increase in cellular ROS (Fig. 1G and H). To further investigate IR-induced cellular membrane damage, we quantified lipid peroxidation—an oxidative cascade that compromises membrane integrity—using C11-BODIPY. We monitored C11-BODIPY fluorescence following 0-8 Gy irradiation at 3, 6, 12, and 24 h post-exposure, with the results presented in Fig. 1E and Fig. S1–S3. Quantitative analysis revealed that C11-BODIPY expression peaked at 6 h after 6 Gy irradiation (Fig. 1E), establishing the radiation conditions for subsequent experiments. Furthermore, the fluorescence intensity of oxidized C11-BODIPY increased significantly with radiation dose (Fig. 1F), confirming that radiation-induced membrane damage resulted from lipid peroxidation.

These results indicated that ferroptosis plays a crucial role in the pathogenesis of RE. Radiation induced ROS production in intestinal epithelial cells, which subsequently elevated lipid peroxidation levels. Ferroptosis, a type of cell death induced by iron-catalyzed ROS generation, primarily operates through lipid peroxidation to cause small intestinal epithelial and colonic cell damage [8,42]. Therefore, the increase of ROS exacerbated the degree of lipid peroxidation, and further promoted the occurrence of ferroptosis, and it was suggested that reducing lipid peroxidation and inhibiting ferroptosis via the clearance of ROS is a promising radioprotective strategy.

### 2.2 Preparation and characterization of EcN-Tyr

To eliminate ROS and inhibit ferroptosis, an engineered probiotic EcN-Tyr was constructed, which was capable of expressing tyrosinase. EcN was selected to construct the delivery system primarily due to its probiotic properties and human biosafety profile [18,20,43]. Moreover, EcN is also an preferred chassis to engineer the expression of different proteins due to its compatibility with current genetic manipulation techniques [18]. The engineered probiotics could biosynthesize melanin through a series of biological cascade reactions. Melanin synthesis initiates with the oxidation of l-tyrosine or L-3,4-dihydroxyphenylalanine (DOPA) to l-DOPA quinone, which are catalyzed by tyrosinase [44,45]. The biosynthesized melanin possesses excellent ROS scavenging abilities, enabling effective ferroptosis inhibition to achieve therapeutic effects.

**Fig. 2:**
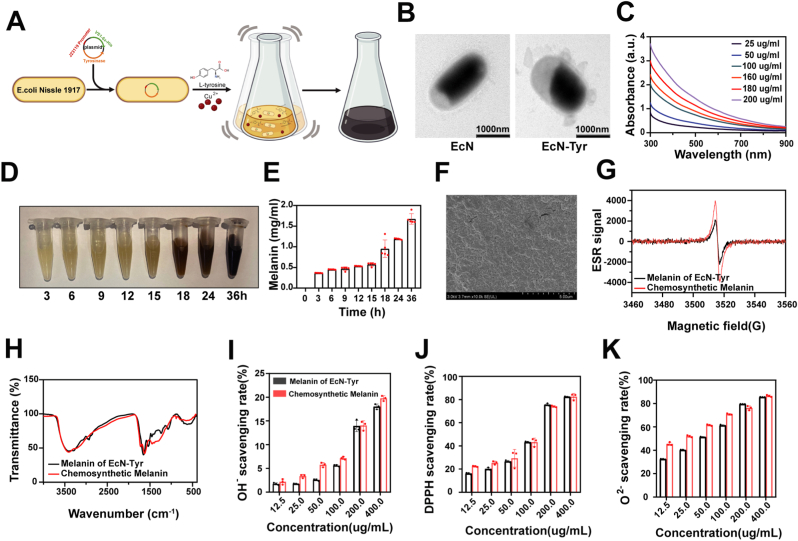
**Characterization of EcN-Tyr and biosynthesized melanin.** A) Scheme of synthesizing melanin from modified EcN. B) Representative TEM images of EcN and EcN-Tyr (Scale bar: 1000 nm). C) The UV–vis-NIR absorption spectra of different concentrations of melanin nanoparticles. D) Photographs were taken at the indicated time points during the biosynthetic process of melanin. E) Quantitative analysis of melanin concentration in LB medium supplemented with CuSO_4_·5H_2_O and l-tyrosine at different time points. Data are presented as mean values ± SEM (n = 5). F) Representative SEM images of biosynthesized melanin produced by EcN-Tyr. G) Electron spin resonance (ESR) spectra of chemically synthesized melanin and biosynthesized melanin. H) Fourier transform infrared (FTIR) spectra of chemically synthesized melanin and biosynthesized melanin. I–K) Scavenging efficiencies of chemically synthesized melanin and biosynthesized melanin against hydroxyl radicals (·OH^−^), DPPH radicals, and superoxide anion radicals (·O_2_^−^), respectively (n = 3).

To endow EcN with biotic melanin production ability, the plasmid containing tyrosinase genes pCm23119 (Fig. S4) was transformed to EcN, yielding the genetically engineered strain EcN-Tyr with stable tyrosinase expression to finally produce biotic melanin (Fig. 2A). Gene modification did not significantly change the morphology of EcN-Tyr, compared with that of EcN, which was shown by transmission electron microscope (TEM) observation (Fig. 2B). To verify that EcN successfully induced melanin production, CuSO_4_·5H_2_O and l-tyrosine were supplemented in Luria-Bertani (LB) medium. The supernatant was collected at the pre-set time point, and the standard curve was plotted using the chemical synthesis product melanin to quantify the melanin concentration. The culture medium changed significantly at 18 h and reached a peak about 1.8 mg mL^−1^ at 36 h (Fig. 2D and E).

Scanning electron microscopy (SEM) images indicated that the melanin produced by EcN-Tyr exhibited a spherical shape with a diameter ranging from 825 to 955 nm (Fig. 2F–S5). The UV-visible absorption spectra of biosynthesized melanin with different concentrations were shown in Fig. 2C. Furthermore, electron spin resonance (ESR) spectroscopy (Fig. 2G) and Fourier transform infrared (FTIR) spectroscopy (Fig. 2H) were performed to compare biosynthesized melanin with chemically synthesized melanin. The results demonstrated that the two exhibited highly similar spectral characteristics, indicating consistent physicochemical properties.

To evaluate the free radical scavenging capacity of biosynthesized melanin, its scavenging effects on hydroxyl radicals (·OH^−^, Fig. 2I), DPPH radicals (Fig. 2J), and superoxide anion radicals (·O_2_^−^, Fig. 2K) were determined. The results indicated that biosynthesized melanin exerted radioprotective effects by scavenging radiation-induced reactive oxygen species (ROS) and reducing free radical damage. Moreover, its protective efficacy was comparable to that of chemically synthesized melanin and showed an obvious concentration-dependent manner. Therefore, the engineered bacterium EcN-Tyr that can produce melanin has been successfully constructed.

### 2.3 Bacterial microspheres coated with sodium alginate and chitosan showed better ability to target inflammatory sites of RE

Oral administration represents an ideal delivery method for treating digestive system diseases. Surface modification of EcN can enhance its tolerance to harsh gastrointestinal conditions—including acidic gastric juice, temperature variations, and osmotic pressure—while also improving targeting specificity and therapeutic efficacy.

Common bacterial surface modifications include microencapsulation (such as layer by layer encapsulation, a multilayer structure formed by electrostatic self-assembly), electrospinning, surface grafting functional groups to bind to various materials and simple mechanical extrusion encapsulation. These approaches aim to strengthen bacterial adhesion, increase gastrointestinal survivability, and achieve more precise targeting and treatment [18].

Earlier research utilized sequential charge-based layering to help EcN withstand the aggressive stomach environment [46]. Once these layers degrade, the negatively charged EcN accumulates at positively charged inflammatory sites, enabling localized therapy [35,47]. However, conventional encapsulation often relies on repeated pH shifts to cross-link sodium alginate, which can substantially compromise bacterial viability. Inspired by prior work, we therefore developed a milder synthesis route for producing alginate hydrogel-coated bacterial microspheres [[48], [49], [50]].

**Fig. 3:**
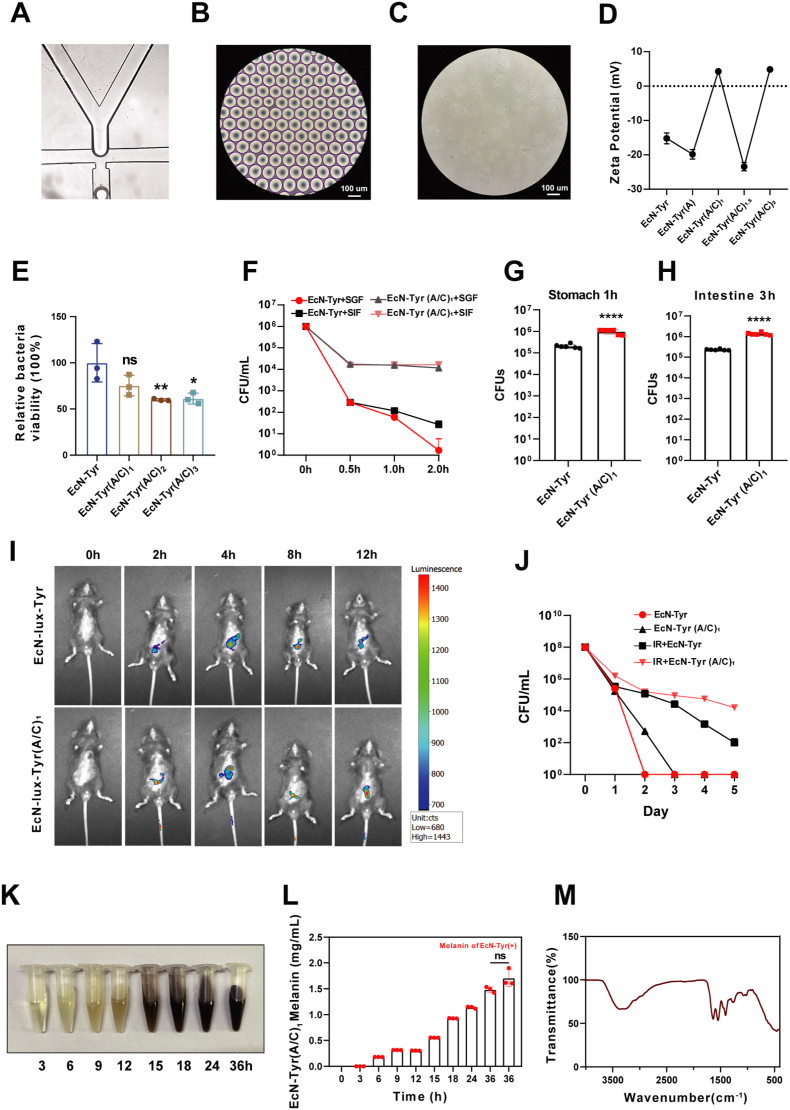
**EcN-Tyr(A/C)_1_ showed better retention in gastrointestinal tract.** A) Synthesis of bacterial hydrogel microspheres produced by microfluidic. B–C) EcN-Tyr (A/C)_1_ gel microspheres were immersed in oil phase and water phase respectively and viewed under an optical microscope (Scale bar: 100 μm). D) Zeta potential of EcN-Tyr with different sodium alginate/chitosan layer coatings. E) The relative viabilities of EcN-Tyr before and after different coatings as indicated. F) Survival quantification of ECN-Tyr and ECN-Tyr (A/C)_1_ exposed to SGF or SIF at 0.5, 1 and 2 h. G-H) Survival quantification of EcN-Tyr and EcN-Tyr (A/C)_1_ 1 h in stomach and 3 h in intestine post-oral gavage. I) In vivo bioluminescence imaging of mice at 0 h, 2 h, 4 h, 8 h, and 12 h after gavage with differently treated bacteria. J) Line graph of bacterial quantification by culture and plating using fecal samples collected from mice of different treatment groups on 0–5 days after gavage. K) Photographs were taken at the indicated time points during the biosynthetic process of melanin produced by EcN-Tyr (A/C)_1_. L) Quantitative analysis of melanin concentration produced by EcN-Tyr (A/C)_1_ in LB medium supplemented with CuSO_4_·5H_2_O and l-tyrosine at different time points, compared with melanin generated by EcN-Tyr at 36h. M) Fourier infrared spectra of biomimetic melanin produced by EcN-Tyr (A/C)_1_. Data are presented as mean values ± SD (n = 3 biologically independent samples for (D, E, K, L), n = 6 biologically independent samples for (F-J)). Statistical analysis was evaluated with two-tailed Student's t tests (*P < 0.05, **P < 0.01, ***P < 0.001, ****P < 0.0001).

As shown in Fig. 3A, aqueous phase 1 containing sodium alginate, Ca-EDTA, combined with EcN-Tyr, and aqueous phase 2 containing Zn-EDDA were joined through a special chip channel and cut by a fast-flowing oil phase to form uniformly distributed microspheres. Fig. 3B and C showed the microspheres immersed in the oil phase and the PBS, respectively. After 30 min of alginate solidification, the pre-prepared chitosan liquor continued to be coated in the outer layer. This process is repeated to produce bacterial microspheres with different layers. Zeta potential analysis confirmed successful encapsulation (Fig. 3D). To conduct quantitative analysis of bacteria in subsequent experiments, we established the linear relationship between EcN and EcN-Tyr with OD600, as shown in Figs. S6 and S7. Meanwhile, we were concerned about that the gel formation might have an impact on bacterial viability. The bacterial vitality with different layers were tested, and it was found that compared with the uncoated EcN-Tyr, the bacterial vitality was significantly inhibited at two layers (Fig. 3E). Therefore, we chose a single-layer EcN-Tyr (A/C)_1_ formulation for further experiments. In order to verify the effectiveness of alginate and chitosan coating in protecting bacteria against gastric acid, we found that more EcN-Tyr (A/C)_1_ bacteria survived after incubation with simulated gastric fluid (SGF) and simulated intestinal fluid (SIF) at different time points in vitro (Fig. 3F–S8, S9). This conclusion was further confirmed in vivo using a mouse model (Fig. 3G and H, S10).

To directly observe the inflammatory targeting efficacy and intestinal retention time of bacterial microspheres, in vivo fluorescence imaging was performed on mice at 0 h, 2 h, 4 h, 8 h, and 12 h after gavage administration. The results showed that the fluorescence signal in the intestine was consistently stronger in the EcN-lux-Tyr (A/C)_1_ group compared with other groups, suggesting longer intestinal retention and superior targeting efficacy (Fig. 3I). To further quantify the intestinal colonization efficiency of bacteria, fecal samples of mice from different treatment groups were collected over 0–5 days after gavage, followed by bacterial culture and quantitative analysis using ampicillin-resistant medium. The results demonstrated that bacteria colonized the intestine more easily after IR irradiation. Moreover, the colonization rate of alginate-chitosan coated EcN-Tyr (A/C)_1_ was significantly higher than that of uncoated EcN-Tyr, confirming that coating modification effectively improved the intestinal colonization ability of engineered bacteria (Fig. 3J).

In order to investigate whether the coating does not affect the synthesis of melanin, we monitored the melanin synthesis process and concentration changes, and characterized the melanin produced by EcN-Tyr (A/C)_1_. The results showed that the coating process did not cause significant changes in melanin particles, compared with the melanin synthesized by EcN-Tyr (Fig. 3K, L, 3M).

### 2.4 EcN-Tyr (A/C)_1_ significantly alleviates RE

**Fig. 4:**
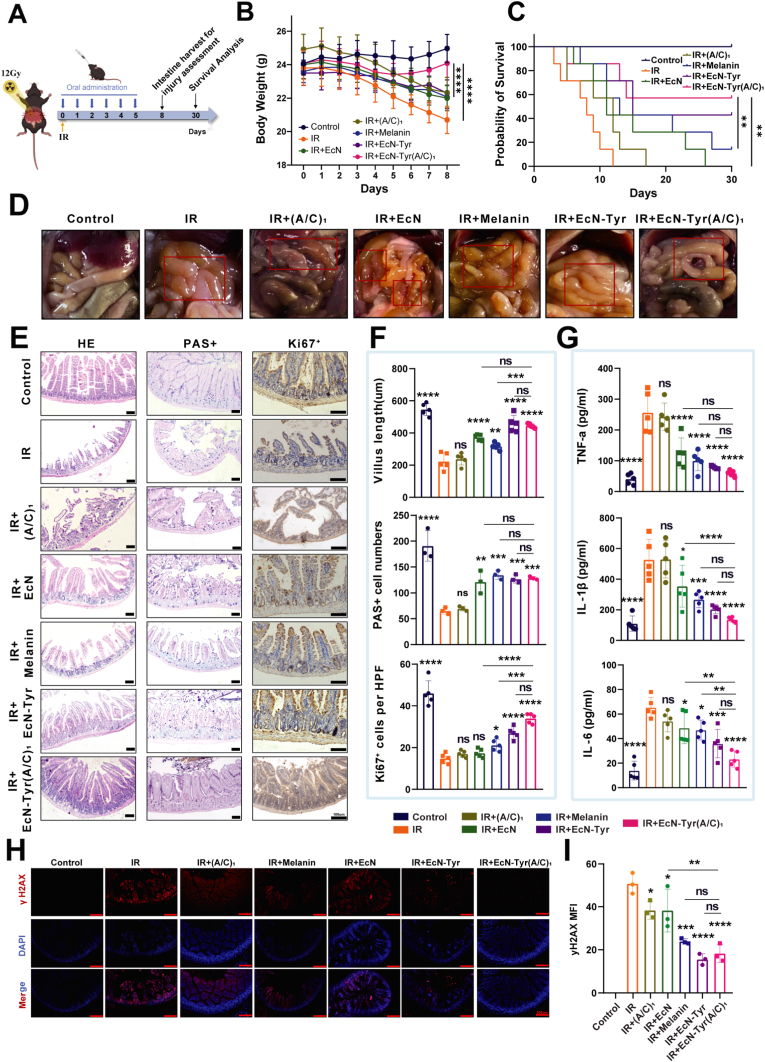
**EcN-Tyr(A/C)_1_ reduces small intestinal damage against IR.** A) Schematic illustration for in vivo radioprotection evaluation. B) Body weight of mice (n = 6). C) Survival curves at day 30 (n = 10), the data were analyzed by Log-rank test. *P* < 0.0001 vs the IR group. D) C57BL/6 mice were gavaged with 10^8^ CFU EcN or EcN-Tyr or 100 mg kg^−1^ melanin 4 h before 12Gy IR. Mice were given PBS only in the control and IR group. Small intestines were collected on day 3 after IR. Photographs of the small intestines that were collected are shown. E) Representative images of intestine by H&E staining and villus length were assessed (n = 5); Representative images of PAS staining and quantification of goblet cells based on PAS staining (n = 3); Representative images of Ki67^+^ in small intestine determined by immunohistochemical (IHC) staining and Ki67-positive cells per crypts were tested quantification based on IHC staining (n = 5. Scale bar: 100 μm). F) Quantitative analysis results of villus length, PAS + staining, and Ki67^+^ staining. G) The levels of TNF-α, IL-1β and IL-6 in the small intestine tissues measured by ELISA (n = 5, respectively). H) Representative immunofluorescence images showing the expression of **γ**H2AX in the small intestines at day 3 post IR. Scale bar: 200 μm. I) The mean fluorescence intensity (MFI) of **γ**H2AX-positive area were tested (n = 3). Data are presented as mean values ± SD (*P < 0.05, **P < 0.01, ***P < 0.001, and ****P < 00001).

To investigate the therapeutic effect of EcN-Tyr (A/C)_1_ on RE, the animal model of RE was established using 12 Gy abdominal IR to mice, followed by a 5-day treatment with different gavage protocols (Fig. 4A). Mice in the treatment group received a gavage of 10^8^ CFU of EcN-Tyr (A/C)_1_, while mice in the (A/C)_1_ group received an equivalent amount of sodium alginate and chitosan. The EcN group received a gavage of 10^8^ CFU of EcN, the melanin group received 100 mg/kg of melanin, and the EcN-Tyr group received a gavage of 10^8^ CFU of EcN-Tyr. On the third day after IR, mice were euthanized and small intestine samples were collected. The results showed that IR-induced intestinal congestion and edema were significantly alleviated by the treatment with EcN-Tyr and EcN-Tyr (A/C)_1_ (Fig. 4D). We continued to monitor the body weight changes of the mice over an 8-day period and found that treatment with EcN-Tyr (A/C)_1_ significantly restored the mice's body weight (Fig. 4B). Moreover, compared with the radiation group, treatment with EcN-Tyr (A/C)_1_ significantly improved the survival rate of mice within 30 days post-exposure to 12 Gy of radiation (Fig. 4C).

The intestinal epithelium is one of the most rapidly self-renewing tissues in the body, making the small intestine the most sensitive and vulnerable part of the gastrointestinal tract to radiation [51]. Intestinal villi are particularly sensitive to radiation. To further explore the impact of EcN-Tyr (A/C)_1_ on small intestinal damage in irradiated mice, we conducted immunohistochemical staining and immunofluorescence analysis of the mouse small intestinal tissue sections. The results indicated that EcN-Tyr (A/C)_1_ significantly reversed the reduction in villus length caused by IR (Fig. 4E). PAS staining analysis showed that EcN-Tyr (A/C)_1_ effectively protected the small intestinal goblet cells of the mice, which function in lubricating the small intestinal epithelial surface and protecting small intestinal epithelial cells from IR-induced damage. The level of Ki67 (a proliferation marker) decreased after radiation, but was significantly elevated in IR mice treated with EcN-Tyr (A/C)_1_, indicating that EcN-Tyr (A/C)_1_ restored the proliferative capacity of small intestinal epithelial cells. Compared with the EcN-Tyr group, EcN-Tyr (A/C)_1_ exhibited similar effects in improving villus length and goblet cell numbers, but was more effective in promoting crypt proliferation (Fig. 4F).

RE led to elevated levels of inflammatory cytokines in the intestinal tissues, including IL-1β, TNF-α, and IL-6 [6]. Studies have shown that EcN regulates inflammation by downregulating the expression of miRNA-155 and miRNA-223 in intestinal tissues, both of which are positively correlated with pro-inflammatory cytokines IL-1β and TNF-α [52]. We found that the levels of inflammatory cytokines such as TNF-α, IL-1β and IL-6 rapidly increased after radiation exposure, but significantly decreased after treatment with EcN-Tyr and EcN-Tyr (A/C)_1_ (Fig. 4G), which was consistent with the previous research [53]. Compared with the EcN-Tyr group, EcN-Tyr (A/C)_1_ was more effective in reducing the levels of TNF-α, IL-1β and IL-6.

Death of terminally differentiated enterocytes is a pathogenic factor in the development of intestinal injury [54]. It is well known that one of the significant IR-induced intracellular damages is due to ROS-induced DNA double-strand breaks [55]. To evaluate DNA double-strand breaks, we assessed γ-H2AX levels via immunofluorescence. Results showed that EcN-Tyr (A/C)_1_ could reduce IR-induced DNA damage in cells (Fig. 4H). However, compared with the EcN-Tyr group, EcN-Tyr (A/C)_1_ did not further improve the DNA repair of intestinal cells in the mice (Fig. 4I).

In summary, EcN-Tyr (A/C)_1_ administration effectively suppressed inflammatory cytokine production linked to IR-induced small intestinal damage, reduced IR-mediated DNA damage in mouse small intestines, promoted the repair of injured small intestinal epithelium, and enhanced small intestinal epithelial cell proliferation. These results demonstrate the efficacy of EcN-Tyr (A/C)_1_ in treating RE.

### 2.5 EcN-Tyr (A/C)_1_ alleviates RE by inhibiting ferroptosis and restoring gut microbiota homeostasis

Next, we sought to elucidate the mechanism underlying the radioprotective function of EcN-Tyr (A/C)_1_. Ferroptosis is implicated in the pathology of numerous intestinal diseases, including intestinal IR injury, IBD, and colorectal cancer [[56], [57], [58], [59], [60]]. Previous study has established ferroptosis as an etiologic factor in radiation colitis, where classic ferroptosis biomarkers increase in correlation with lesion site severity [61]. Unlike apoptosis, pyroptosis, and autophagy, ferroptosis is an iron-dependent form of regulated cell death driven by an imbalance between oxidants and antioxidants [62]. These observations suggest that specifically targeting ferroptosis could offer a viable therapeutic strategy for IR-induced intestinal injury.

**Fig. 5:**
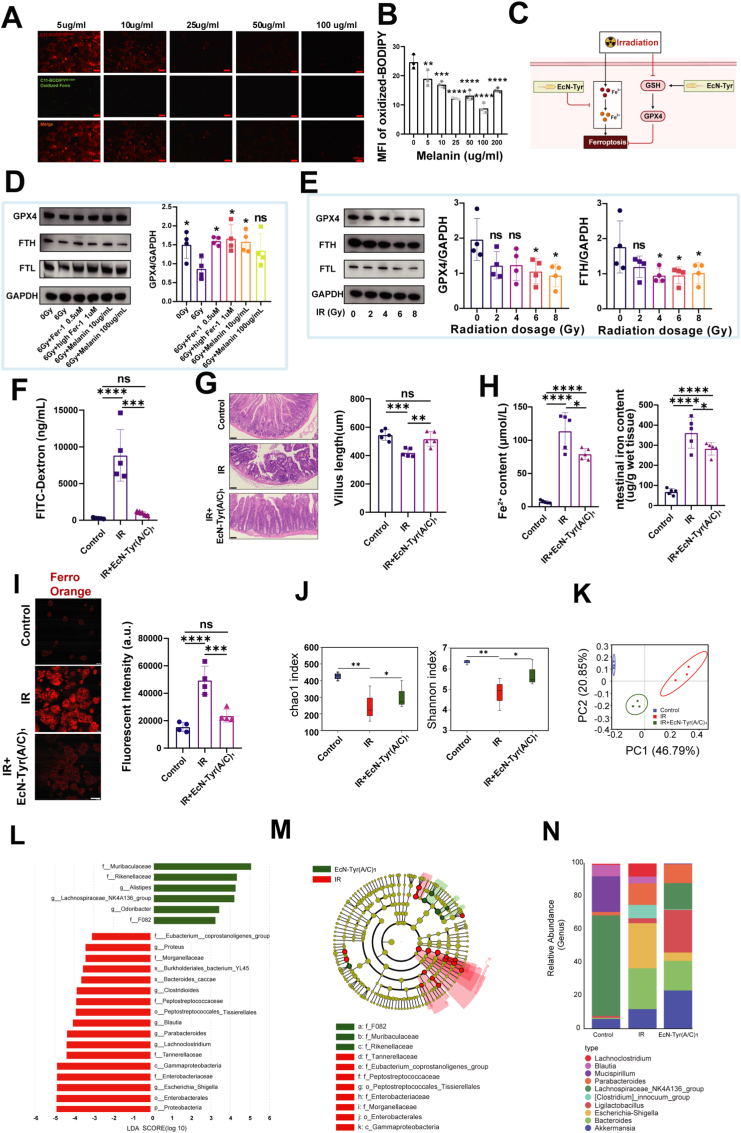
**EcN-Tyr(A/C)_1_ alleviates IR-induced small intestinal damage in mice by inhibiting ferroptosis and rebalancing the gut microbiota.** A) Fluorescence images of reduced (red) and oxidized (green) C11-BODIPY of Caco-2 and B) their mean fluorescence intensity quantification (n = 3. scale bars: 100 μm). C) Schematic diagram of ferroptosis regulation. D) After treatment with melanin or iron inhibitors, the expression levels of ferroptosis-related proteins GPX4 in Caco-2 cells were analyzed using Western blotting (with GAPDH as a loading control), and representative images were obtained (n = 4). E) After IR with different doses, the expression levels of ferroptosis-related proteins GPX4 and FTH in Caco-2 cells were analyzed using Western blotting (with GAPDH as a loading control), and representative images were obtained (n = 4). F) Quantitative analysis of intestinal FITC-Dextran permeability in mice of different treatment groups (n = 5). G) Reresentative images of intestine by H&E staining and villi length was assessed (n = 5. Scale bar: 100 μm). H) Histogram of mouse small intestinal iron quantification analysis (n = 5). I) Fluorescent images and quantitative analysis of Fe^2+^ in cells of different treatment groups by FerroOrange staining (n = 4. Scale bar: 50 μm). J) Chao1 and Shannon index of microbial community (n = 3). K) Principal component analysis (PCA) of gut microbiome (n = 3). L-M) Linear discriminant analysis Effect Size (LEfSe) analysis of the microbiota in different treatments (n = 3). N) Community bar plot analysis of the microbiota of mice at the genus level (n = 3). Data are shown as mean ± SD. One - way ANOVA was used for overall multiple - group analysis, and Tukey's post - hoc test was applied when ANOVA showed significant differences. Significance levels are marked as follows: *P < 0.05, **P < 0.01, ***P < 0.001, ****P < 0.0001.

In the in vitro experiments, Caco-2 cells were exposed to 6 Gy of IR and subsequently treated with melanin secreted by engineered bacteria at concentrations ranging from 5 to 200 μg/mL. The results showed that as the melanin concentration increased, and the fluorescence signal of the lipid peroxidation indicator C11-BODIPY gradually decreased (Fig. 5A). However, the ability of melanin to scavenge lipid peroxides were not further enhanced with the higher concentrations. The inhibitory effect reached its maximum at a melanin concentration of 100 μg/mL (Fig. 5B), which might be related to the cytotoxicity of melanin (Fig. S11). Compared with the group exposed to 6 Gy of radiation, the application of the ferroptosis inhibitor Fer-1 led to a decrease in the fluorescence signal of C11-BODIPY, and similar results were observed under the intervention of melanin at concentrations of 10 and 100 μg/mL (Fig. S12).

GPX4 is widely recognized as the principal inhibitor of ferroptosis. Iron metabolism is closely linked to proteins such as FTH and FTL, which store and detoxify intracellular iron [63]. Alterations in these proteins confirm the dysregulation of iron metabolism following radiation exposure [64]. Elevated FTH expression helps prevent intracellular iron overload and thereby reduces cellular ferroptosis. The study found that both the Fer-1 inhibitor and melanin intervention primarily targeted GPX4, with no significant effects on FTH and FTL (Fig. 5D–S13). To further explore the ferroptosis signaling pathway, Caco-2 cells were exposed to a gradient of radiation from 0 to 8 Gy. The results indicated that GPX4 and FTH were significantly reduced at 6 Gy radiation exposure, while no significant changes were observed in FTL (Fig. 5E–S14).

In previous in vivo experiments, we confirmed the effectiveness of the engineered bacterium microspheres EcN-Tyr (A/C)_1_ in protecting against RE. To further investigate the mechanism of EcN-Tyr (A/C)_1_ in the RE treatment, we exposed C57BL/6 mice to 12 Gy of radiation and subsequently treated with EcN-Tyr (A/C)_1_ for 5 days. Intestinal mucosal barrier function was then detected by using the FITC-Dextran assay. As shown in Fig. 5F, compared with the IR group, the EcN-Tyr (A/C)_1_ treatment group significantly improved intestinal mucosal barrier function in mice. Mice were euthanized on day 8, mice were euthanized and small intestine samples were collected. The results showed that EcN-Tyr (A/C)_1_ effectively protected mice from acute intestinal injury induced by IR, as evidenced by the significantly preserved length of small intestinal villi (Fig. 5G). The activity of GPX4 depends on reduced glutathione (GSH), and the depletion of GSH or the inactivation of GPX4 can trigger ferroptosis through the accumulation of intracellular lipid ROS and excessive lipid peroxidation [65]. Here, we found that the expression levels of GPX4 and GSH in mouse small intestinal tissue decreased after IR (Figs. S15 and S16), while the levels of oxidized glutathione (GSSG) significantly increased (Fig. S17), and the oral treatment with EcN-Tyr (A/C)_1_ could effectively reverse these pathological changes.

Beyond the lipid peroxidation pathway, iron metabolism also critically regulates ferroptosis. Excess iron promotes ferroptosis through the Fenton reaction [65]. Within endosomes, Fe^3+^ imported via the transferrin receptor (TR) is reduced to Fe^2+^ by the metalloreductase Steap3 and subsequently released into the cytosol by divalent metal transporter 1 (DMT1) [66]. As shown in Fig. 5H and S18, the levels of Fe^2+^ and total iron in mouse small intestinal tissue were significantly increased after IR, but were markedly decreased after treatment with EcN-Tyr (A/C)_1_. To further verify these results, we detected intracellular Fe^2+^ in Caco-2 cells by using FerroOrange staining, and the findings were consistent with the above in vivo results (Fig. 5I–S19). These results indicated that EcN-Tyr (A/C)_1_ mitigated IR-induced intestinal damage by regulating ferroptosis, which is a process of cell death caused by abnormal intracellular iron metabolism (Fig. 5C).

Previous reports have established that IR triggers intestinal dysbiosis, altering microbial composition, functionality, and metabolite profiles [10,53]. Therefore, 16S rRNA sequencing analysis of fecal samples was further conducted to investigate the alterations in gut microbiota in different groups. The results showed that the EcN-Tyr (A/C)_1_ treatment group significantly increased the Chao1 and Shannon diversity indices, thereby enhancing microbial diversity (Fig. 5J). Principal coordinates analysis (PCoA) indicated that, compared with the RE model group, the EcN-Tyr (A/C)_1_ intervention significantly affected the overall microbial composition (Fig. 5K). Linear discriminant analysis effect size (LEfSe) revealed that there were significant changes in the microbial community (LDA score >3) (Fig. 5L and M). Further analysis at the genus level indicated that the EcN-Tyr (A/C)_1_ treatment group achieved therapeutic effects by increasing the abundance of beneficial bacteria, such as Akkermansia and Ligilactobacillus, while reducing the abundance of harmful bacteria, such as Escherichia-Shigella (Fig. 5N). These results supported that EcN-Tyr (A/C)_1_ exerts therapeutic effects by beneficially modulating the balance of gut microbiota.

### 2.6 Biosafety of engineered EcN-Tyr (A/C)_1_

**Fig. 6:**
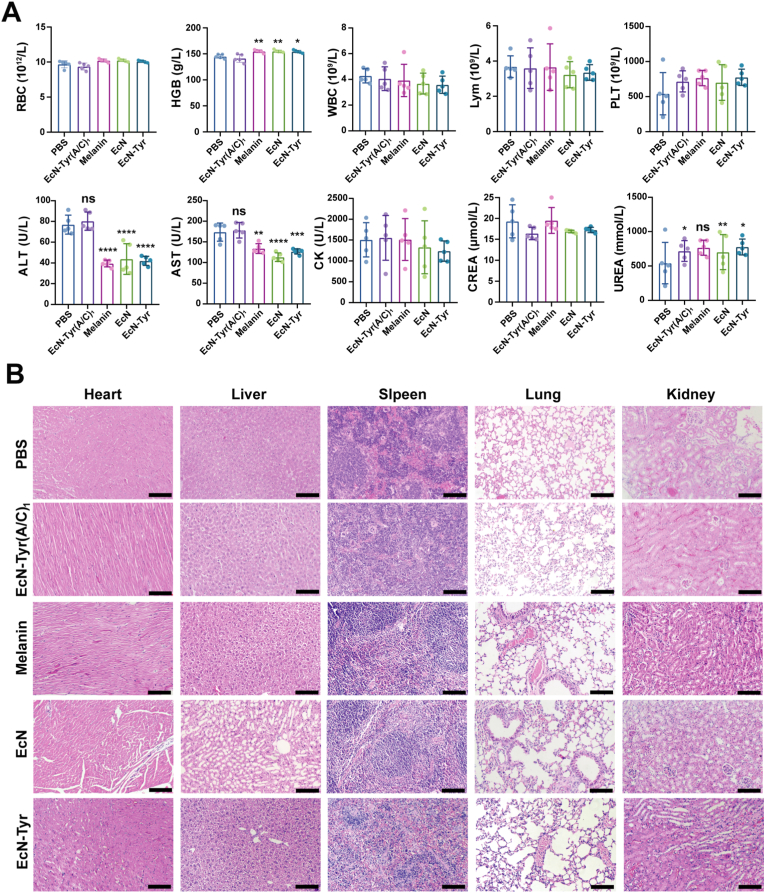
**The biosafety of EcN-Tyr(A/C)**_1_**.** A) Serum biochemical indexes and B) H&E staining and of the main organs of the mice at 14 days post-treatment (scale bars: 100 μm). Data were showed as mean ± SD (n = 5 per group. *P < 0.05, **P < 0.01, ***P < 0.001, and ****P < 00001). The data were analyzed by one-way ANOVA with Tukey's post hoc test compared with the PBS group.

Encouraged by the effective therapeutic results achieved by EcN-Tyr (A/C)_1_ in the RE model, we then determined the in vivo biosafety of EcN-Tyr (A/C)_1_. In these experiments, serum biochemistry assays and complete blood panel tests were performed on day 14 following repeated oral administrations of EcN-Tyr (A/C)_1_ (Fig. 6A). As anticipated, the blood cell - related indicators in mice following EcN-Tyr (A/C)_1_ treatment, encompassing white blood cells, red blood cells, hemoglobin levels, and platelet counts, were in line with those observed in healthy mice. Similarly, the parameters reflecting liver and kidney function, such as alanine aminotransferase (ALT), aspartate aminotransferase (AST), creatine kinase (CK), and creatinine (CREA), all fell within the normal physiological ranges.HE sections of the heart, liver, spleen, lung, and kidney further confirmed the safety of EcN-Tyr (A/C)_1_ (Fig. 6B). All of these results revealed that EcN-Tyr (A/C)_1_ did not induce any adverse effects, and showed satisfactory biosafety.

## 3 Discussion

This study presents a novel therapeutic strategy for RE by constructing the engineered bacterium EcN-Tyr and formulating it into orally administrable microspheres, EcN-Tyr (A/C)_1_. Here, we found that ferroptosis plays a significant role in the development of RE, and EcN-Tyr (A/C)_1_ could effectively mitigate IR-induced small intestinal injury via ferroptosis inhibition. Specifically, EcN-Tyr (A/C)_1_ alleviates small intestinal hyperemia and edema, protects the length of small intestinal villi, crypt proliferation and small intestinal goblet cell count, and reduces DNA damage and inflammatory cytokine levels.

From a mechanism standpoint, EcN-Tyr regulated ferroptosis-related indicators (such as GPX4, GSH and iron content) to reduce lipid peroxidation and iron metabolism disorders, thereby exerting protective effects. The experimental results showed that mice treated with EcN-Tyr (A/C)_1_ exhibited significant weight gain after radiation exposure, a higher survival rate and marked alleviation of DNA damage in small intestinal tissues. Additionally, EcN-Tyr (A/C)_1_ significantly reversed the dysbiosis caused by RE, and restored the balance of gut microbiota by increasing the abundance of beneficial bacteria and concurrently reducing the number of harmful bacteria, thereby further improving the overall health of the intestine.

Notably, the superior efficacy of EcN-Tyr (A/C)_1_ over individual components (EcN alone or melanin alone) stems from its synergistic composite system. The sodium alginate-chitosan coating enables EcN-Tyr to withstand gastric acid, target injured small intestinal mucosa, and achieve in-situ sustained melanin synthesis—continuously scavenging ROS and inhibiting ferroptosis. Meanwhile, EcN itself modulates gut microbiota by enriching beneficial bacteria (e.g., Akkermansia, Ligilactobacillus) and reducing harmful ones (e.g., Escherichia-Shigella), alleviating inflammation and strengthening the intestinal barrier. This “targeted delivery + in-situ melanin synthesis + microbiota regulation” synergy achieves 1 + 1>2 therapeutic effects, outperforming single-agent interventions.

The biosafety of EcN-Tyr (A/C)_1_ was also evaluated in this study. No adverse effects on hematological and biochemical parameters, nor significant histological abnormalities in mice, were observed. These findings demonstrated the favorable biosafety profile of EcN-Tyr (A/C)_1_, making it suitable for in vivo therapeutic applications.

In summary, the developed EcN-Tyr (A/C)_1_ acts as a multimodal therapeutic platform that simultaneously delivers melanin-based radioprotection, suppresses ferroptosis, repairs epithelial DNA damage, suppresses inflammation, and restores a beneficial gut microbiota composition. This integrated therapeutic efficacy, together with its demonstrated safety, establishes a solid translational basis for clinical use against RE.

## 4 Materials and methods

### 4.1 Strain construction

To construct the *Escherichia coli* Nissle 1917 (EcN-Tyr) strain, 1 μL of the pCm23119 plasmid (provided by Forhigh Biotech Hangzhou) was added to electrocompetent cells and incubated on ice for 5 min. Following this, electroporation was performed at 2500 V. After electroporation, 1 mL of LB medium was added, and the cells were incubated at 37 °C for 1 h. The culture was then spread onto LB agar plates containing 34 mg mL^−1^ chloramphenicol, and the resulting colonies were inoculated into 100 mL of LB liquid medium with chloramphenicol resistance, followed by incubation at 37 °C. Finally, the strain was stored in sterile 50% glycerol at −80 °C.

### 4.2 Melanin synthesis and characterization

To observe melanin generated by EcN-Tyr, 50 μg mL^−1^ CuSO_4_·5H_2_O solution, and 0.8 mg mL^−1^ l-tyrosine were added to the LB broth medium incubated at 37 °C with shaking (150 rpm). The supernatant was extracted at different time points to detect the melanin concentration according to standard concentration curve. Size distributions were measured using Nano ZS90 Laser Particle Size Analyzer (Malvern, UK). Melanin was imaged using a Scanning electron microscope (SEM; HIYACHI MC1000, Japan).

### 4.3 Preparation of bacterial microspheres and EcN-Tyr (A/C)_n_

Bacterial microspheres were generated according to the ion exchange method. In brief, liquid phase 1 (40 mM Calcium-EDTA and 0.6 mg mL^−1^ sodium alginate containing EcN-Tyr) were mixed with liquid phase 2 (40 mM Zn-EDDA solution), and then were entrapped again in an oil phase to form sodium alginate gel microspheres named EcN-Tyr(A). The flow rate of the oil phase is varied to adjust the size of the droplets through Three-channel Microdroplet Preparator and 100 μm microfluidic chip (FluidicLab, Shanghai, China). The demulsifier was used to remove oil on the microspheres' surface and washed with PBS three times. Then, EcN-Tyr(A) was resuspended in chitosan solution (2 mg mL^−1^, PH 6.0) for 30 min and obtained EcN-Tyr (A/C)_1_. After washing twice with PBS, the coating steps were repeated for the second coating layer. The zeta potentials of EcN-Tyr, EcN-Tyr(A), EcN-Tyr (A/C)_1_, EcN-Tyr (A/C)_1.5_ and EcN-Tyr (A/C)_2_ were detected following instructions of Nano ZS90 Laser Particle Size Analyzer (Malvern, UK).

### 4.4 Bacterial viability assay

To analyze the viability of EcN-Tyr with different chitosan/sodium alginate coatings, 190 μL EcN-Tyr, EcN-Tyr (A/C)_1_, EcN-Tyr (A/C)_2_ and EcN-Tyr (A/C)_3_ were added to the 96-well plate, 10 μL CCK-8 solution was added, and the mixture was cultured at 37 °C for 4 h. The viability of bacteria was assessed by the OD value of the mixture solution at 450 nm.

### 4.5 Resistance analysis of EcN-Tyr (A/C)_1_ in vitro and in vivo

1 × 10^6^ CFU EcN-Tyr or EcN-Tyr (A/C)_1_ were resuspended in PBS (Control), SGF or SIF at 37 °C with gentle shaking for 2 h. Then, the bacteria in each sample were collected at different time points by centrifugation, washed and diluted with PBS, and 100 μL of each diluted sample was spread on solid agar plates. The number of bacteria was determined by the number of colonies after 48 h of incubation in microbiological incubators. To detect the survival of engineered probiotics in the GI tract, male C57BL/6 mice were gavaged with 1 × 10^8^ CFU EcN-Tyr or EcN-Tyr (A/C)_1_. Then, the mice were sacrificed, and contents in their stomach, intestine were collected, homogenized, and diluted with PBS. The dilution factor was consistent at 10%. In total, 100 μL of each diluent was spread on a solid agar plate and cultured in a microbiological incubator for 48 h, and then the colonies of each plate were counted. To further determine the survival of probiotics in the digestive tract, male C57BL/6 mice were gavaged with EcN-lux or EcN-lux (A/C)_1_ (1 × 10^8^ CFU), and then mice and their GI tracts were imaged using the IVIS Imaging System (PerkinElmer, Lumina III).

### 4.6 Cell lines and cell culture

Caco-2 cell line (human colonic epithelial cells) was obtained from the American Type Culture Collection (ATCC, Manassas, Virginia, USA), which was cultured under the condition of a humidified atmosphere (5% CO_2_) at 37 °C. The complete medium for culture consisted of 90% a-MEM medium, 20% fetal bovine serum, 1% NEAA and 1% penicillin-streptomycin. The operating parameters of X-ray IR were set at 2, 4, 6, 8 Gy (RadSource Technologies Inc RS2000pro-225, America).

### 4.7 Cellular ROS and lipid peroxidation detection

Cells were seeded in 3.5 cm plates 24 h prior to treatment, pretreated with 0.5 or 1 μM Fer-1 (MCE, USA), or 10, 100 μg mL^−1^ melanin for 6 h. Detection of cellular ROS was based on the peroxide-dependent oxidation of DCFH-DA (Beyotime, China) to form a fluorescent compound named dichlorofluorescein (DCF). Cellular ROS were immediately analyzed by flow cytometry (Canto, BD Biosciences, USA). 6 h after IR, cells were washed with PBS and incubated with 1 μM DCFH-DA at 37 °C for 30 min. For lipid peroxidation assay, fresh medium containing 5 μM C11-BODIPY dye (D3861, Invitrogen, USA) was added to each well 6 h after IR, and the cells were incubated at 37 °C for 30 min. The cells were washed with PBS and trypsinized to obtain a cell suspension. Lipid peroxidation levels were tested by immunofluorescence. The visual image of the DCF and C11-BODIPY fluorescence in cells was taken on fluorescence microscopy.

### 4.8 Animals

C57BL/6 male mice (6-8 weeks old) were bought from Beijing Vital River Laboratory Animal Technology Co., Ltd. (Beijing, China). They were kept in a SPF animal facility with a temperature of 24 ± 1 °C, humidity of 51 ± 5%, and a 12-h light/12-h dark cycle. The mice were grouped in cages of five to six per cage and had free access to food and water, ensuring animal welfare. All animal procedures were approved by Laboratory Animal Welfare and Ethics Committee of Army Medical University, Chongqing, China (AMUWEC20247001).

### 4.9 IR-induced enteritis model and treatment

An X-ray exposer (RadSource Technologies Inc RS2000pro-225, America) was used for all experiments. To study IR-induced enteritis, except for the control, all other groups were exposed to 12 Gy of X-ray, the operating parameters were: 159.2 kV/24.8 mA energy, two 5-cm-thick lead plates were used to avoid whole-body exposure. Mice were subjected to whole abdominal IR of 12 Gy. The mice were randomly grouped, including the control group (100 μL of saline), IR group (100 μL of saline), IR+(A/C)_1_ group (0.06 mg sodium alginate and 0.1 mg chitosan solutions), IR + EcN group (10^8^ CFU), IR + Melanin group (100 mg kg^−1^), IR + EcN-Tyr group (10^8^ CFU), IR + EcN-Tyr (A/C)_1_ group (0.06 mg sodium alginate and 0.1 mg chitosan solutions containing 10^8^ CFU EcN-Tyr). The mice were orally administrated 4 h before IR. Mice were dosed daily for 5 days. During the whole therapeutic period, the body weight of the mice was recorded every day. The mice were euthanatized, and the small intestines were collected at the end of the treatment period. The above tissue was washed with saline and fixed in 4% paraformaldehyde for HE staining. Animals in the survival analysis groups received the same treatment as described above, and their survival was observed for 30 days to assess survival status.

### 4.10 Intestinal ferroptosis measurements

Then Tissue Iron Content Assay Kit (Solarbio, China) and Ferrous Ion Content Assay Kit (BC5415, Solarbio, China) were used to detect the intracellular Fe content and the samples were measured using a microplate reader.

### 4.11 Immunohistochemistry and immunofluorescence

For ki67 and **γ**H2AX, immunohistochemical hypersensitivity UltraSensitiveTM SP kit (Fuzhou Maxim, China) was used according to manufacturer's instructions. 4% paraformaldehyde-fixed paraffin-embedded small intestine sections were deparaffinized, rehydrated, antigen-retrieved for 3 min in citrate buffer (0.1 M, pH 6) and peroxidase-blocked. Small intestine sections were incubated with anti-Ki67 (1:100, proteintech, USA) diluted in goat serum solution overnight at 4 °C. Next, sections were incubated with Biotin-labeled sheep anti-rabbit secondary antibodies and 3,3′-diaminobenzidine. Images were acquired by Olympus BX43 microscope (Olympus America, Center Valley, PA, USA). Integrated optical density was used to evaluate the protein immunoreactivity by ImageJ 1.8.0. Small intestine injury was observed using immunofluorescence. Tissues harvested from the small intestine were fixed with 4% paraformaldehyde, embedded, and cryosectioned to obtain 4 μm sections. After routine dewaxing, rehydration, antigenic repair with sodium citrate at high temperature and pressure, permeabilization (0.1% Triton X-100/PBS), and blocking with quick blocking buffer (Beyotime, China), sections were incubated with primary antibodies **γ**H2AX,1:50 (abclonal, China) at 4 °C overnight. The secondary antibody was Alexa Fluor 594 Goat Anti-Rabbit IgG (h + L) (1:200, abclonal, China). Sections were washed three times with PBS to remove non-specific binding. Following the previous step, the tissue sections were incubated with secondary antibodies at ambient temperature for 2 h.After incubation, sections were then counterstained using 4′,6-diamidino-2-phenylindole (DAPI) for 10 min and mounted in an antifade mounting medium (Beyotime, China).

### 4.12 Periodic acid-Schiff (PAS) staining

PAS staining was used to observe small intestinal mucin staining for goblet cell function and mucus barrier estimation. Briefly, 3-μm-thick sections were stained using a PAS staining kit (Solarbio, China) and counterstained with hematoxylin according to the instructions from the manufacturer.

### 4.13 Small intestinal tissue inflammation analysis

Elisa kits (Solarbio, Beijing, China) were used to detect the levels of IL-1β, IL-6 and TNF-α in supernatants according to manuals.

### 4.14 Western blot

The total protein levels were detected by BCA protein assay kits (Beyotime, Shanghai, China). In brief, the supernatant of cells and mice brain tissues was lysed with radio immuno-precipitation assay (RIPA) buffer containing phenylmethyl sulfonyl fluoride (PMSF) and centrifuged at 12000 rpm for 10 min. Protein concentrations were measured using BCA kits. Protein samples in a mixture, with a volume of 20 μL loaded per lane, were resolved via sodium dodecyl sulfate - polyacrylamide gel electrophoresis (SDS - PAGE). Subsequently, the separated proteins were transferred onto polyvinylidene difluoride (PVDF) membranes (pore size: 0.45 μm, purchased from Millipore).Membranes were blocked with 5% nonfat dry milk for 2 h at room temperature. Determining the concentrations of protein, the cell extraction incubated with primary antibody was performed overnight at 4 °C. The following primary antibodies were used: GPX4 (proteintech, 1:1000, USA), FTH CST, 1:1000, USA), FTL (proteintech, 1:1000, USA), β-actin (abcam, 1:1000, USA). The next day, the membranes were washed 3 times for 5 min each with TBST and then incubated with HRP-conjugated secondary antibody (1:10,000 dilution) for 2 h at room temperature. Western Blot signals were detected by chemiluminescence with Chemidoc software (Bio-Rad, Munich, Germany).

### 4.15 Biosafety evaluation of the microsphere in vivo

After 14 days of treatment (1 × 10^8^ CFU EcN/EcN-Tyr), the mice were sacrificed, and the major organs were collected and stored in 4% paraformaldehyde buffer for H&E staining. Additionally, blood samples were collected from the Orbital vein (500 μL each mouse), respectively. The blood biochemistry analysis was measured by a biochemical autoanalyzer (Type 7170, Hitachi, Japan).

### 4.16 Statistical analysis

T-test and one-way ANOVA were used for statistical analysis (Graphpad Prism 8). Data are presented as mean ± standard deviation (SD). Statistical differences were defined as ****P < 0.0001, ***P < 0.001, **P < 0.01, *P < 0.05 and ns means no significance.

## CRediT authorship contribution statement

**Chaoqun Lv:** Conceptualization, Data curation, Formal analysis, Investigation, Methodology, Project administration, Resources, Software, Supervision, Validation, Visualization, Writing – original draft. **Hongqing Li:** Conceptualization, Data curation, Formal analysis, Investigation, Methodology, Project administration, Resources, Software, Supervision, Validation, Visualization, Writing – original draft. **Xiang Li:** Conceptualization, Data curation, Formal analysis, Investigation, Methodology, Project administration, Resources, Software, Supervision, Validation, Visualization, Writing – original draft. **Wen Shi:** Conceptualization, Data curation, Formal analysis, Investigation, Methodology, Project administration, Resources, Software, Supervision, Validation, Visualization, Writing – original draft. **Wenbo Li:** Conceptualization, Data curation, Formal analysis, Investigation, Methodology, Project administration, Resources, Software, Supervision, Validation, Visualization, Writing – original draft. **Zhenxing Li:** Data curation, Formal analysis, Investigation, Methodology, Software, Validation, Visualization, Writing – original draft. **Xinyue Hu:** Data curation, Formal analysis, Investigation, Methodology, Software, Validation, Visualization, Writing – original draft. **Xinxin Liu:** Data curation, Formal analysis, Investigation, Methodology, Software, Validation, Visualization, Writing – original draft. **Yuanyuan Ai:** Data curation, Formal analysis, Investigation, Methodology, Software, Validation, Visualization, Writing – original draft. **Zhipeng Wen:** Data curation, Formal analysis, Investigation, Methodology, Resources, Software, Validation, Visualization, Writing – original draft. **Feng Liu:** Data curation, Formal analysis, Investigation, Methodology, Validation, Visualization, Writing – original draft. **Yi Ru:** Data curation, Formal analysis, Investigation, Methodology, Validation, Visualization, Writing – original draft. **Haijun Xiao:** Data curation, Formal analysis, Investigation, Methodology, Validation, Visualization, Writing – original draft. **Jingchao Li:** Conceptualization, Formal analysis, Funding acquisition, Investigation, Methodology, Project administration, Resources, Supervision, Writing – review & editing. **Xiao Chen:** Conceptualization, Formal analysis, Funding acquisition, Investigation, Methodology, Project administration, Resources, Supervision, Writing – review & editing. **Kaijun Liu:** Conceptualization, Formal analysis, Funding acquisition, Investigation, Methodology, Project administration, Resources, Supervision, Writing – review & editing.

## Declaration of competing interest

The authors declare that they have no known competing financial interests or personal relationships that could have appeared to influence the work reported in this paper.

## Data Availability

Data will be made available on request.
