# Supplementary material for: Genetically engineered Escherichia coli Nissle 1917 enabling on-site melanin synthesis attenuates radiation enteritis through ferroptosis inhibition and gut microbiota modulation
Source: Redox Biol. 2026 Mar 25;92:104141. doi: 10.1016/j.redox.2026.104141 (PMC13054290; doi:10.1016/j.redox.2026.104141)
Supplement: Multimedia component 1 [file mmc1.doc]

S1: Fluorescence images of reduced (red) and oxidized (green) C11-BODIPY in Caco-2 cells after 3 hours of IR, along with the quantified results of average fluorescence intensity (n=5. scale bar: 100 μm).

S2: Fluorescence images of reduced (red) and oxidized (green) C11-BODIPY in Caco-2 cells after 12 hours of IR, along with the quantified results of average fluorescence intensity (n=5. scale bar: 100 μm).

S3: Fluorescence images of reduced (red) and oxidized (green) C11-BODIPY in Caco-2 cells after 24 hours of IR, along with the quantified results of average fluorescence intensity (n=5. scale bar: 100 μm).

S4: Plasmid atlas of pCm23119-tyr which carried tyrosinase gene Tyr derived from bacillus megaterium.

S5: Hydrodynamic size distribution of melanin nanoparticles measured by DLS.

S6: The linear relationship between EcN and OD600.

S7: The linear relationship between EcN-Tyr and OD600.

S8: In vitro incubation images of EcN-Tyr with SGF and SIF at different time points.

S9: In vitro incubation images of EcN-Tyr(A/C)_1_ with SGF and SIF at different time points.

S10: In vivo incubation images of EcN-Tyr / EcN-Tyr(A/C)_1_ with SGF and SIF at different time points.

S11: Detection of cell toxicity in Caco-2 cells using different concentrations of melanin (n=5).

S12: Immunofluorescence images of reduced (red) and oxidized (green) C11-BODIPY and their mean fluorescence intensity quantification (n=3. scale bars: 100 μm).

S13: After treatment with melanin or iron inhibitors, the expression levels of ferroptosis-related proteins FTH and FTL in Caco-2 cells were analyzed using Western blotting (with GAPDH as a loading control) (n=4).

S14: After IR with different doses, the expression levels of ferroptosis-related proteins FTL in Caco-2 cells were analyzed using Western blotting (with GAPDH as a loading control) (n=4).

S15: Representative Western blotting image of GPX4 and analysis in small intestinal tissue of mice (relative to GAPDH levels) (n=4).

S16: GSH levels in intestinal tissues (n=5).

S17: GSSG levels in intestinal tissues (n=5).

S18: FerroOrange staining of colonic tissues in mice of different treatment groups (scale bars: 50 μm).

.

S19: Flow cytometric quantitative analysis of Fe²⁺ in cells of different treatment groups by FerroOrange staining (n=4).
